# Short-term changes in tear osmolarity after instillation of different osmolarity eye drops in patients with dry eye

**DOI:** 10.1038/s41598-023-35965-0

**Published:** 2023-07-07

**Authors:** Woojin Kim, In Ho Woo, Youngsub Eom, Jong Suk Song

**Affiliations:** 1 Department of Ophthalmology, Korea University Guro Hospital, 148, Gurodong-Ro, Guro-Gu, Seoul, Republic of Korea; 2 Department of Ophthalmology, Korea University Ansan Hospital, 123, Jeokgeum-Ro, Danwon-Gu, Ansan-Si, Gyeonggi-Do Republic of Korea

**Keywords:** Conjunctival diseases, Corneal diseases

## Abstract

This study investigated short-term changes in tear osmolarity of dry eye patients after using artificial tears containing sodium hyaluronate (SH) at different osmolarities. It comprised 80 patients with dry eye whose tear osmolarity measurement using the TearLab osmolarity system was 300 mOsm/L or greater. Patients who had external ocular disease, glaucoma, or other concomitant ocular pathology were excluded. After being randomly divided into four groups, the participants received different kinds of SH eye drops as follows: Groups 1–3 were given one of three concentrations (0.1%, 0.15%, and 0.3%) of isotonic drops, while Group 4 received 0.18% hypotonic SH eye drops. The tear osmolarity concentrations were evaluated at baseline and again at 1-, 5-, and 10-min after instillation of each eye drop. Tear osmolarity showed a significant decrease after instillation of four types of SH eye drops after up to 10 min compared to baseline. Patients who received hypotonic SH eye drops showed an enhanced decrease in tear osmolarity compared with the isotonic SH eye drops after 1 min (p < 0.001) and 5 min (p = 0.006), but the difference was not significant at 10 min (p = 0.836). The enhanced immediate effect of hypotonic SH eye drops at lowering tear osmolarity in patients with dry eye seems to be limited unless these drops were used frequently.

## Introduction

Dry eye syndrome is a multifactorial disease in which loss of tear film homeostasis plays a central role in pathophysiology^1^. Increased osmolarity of the tear film and inflammation of the ocular surface are also involved^2,3^, and increasing evidence suggests that tear hyperosmolarity plays a pivotal role in pathogenesis of dry eye^4–6^. Tear hyperosmolarity increases the levels of proinflammatory cytokines and chemokines that stimulate a series of inflammatory events in the corneal epithelium^7^. These inflammatory events lead to apoptosis of the epithelial cells, including goblet cells, and eventually to worsening of the vicious cycle^8^.

Several hypotonic tear substitutes have been developed that focus on the central role of tear hyperosmolarity in the pathogenesis of dry eye syndrome and are expected to reduce tear osmolarity and restore osmotic balance. A few studies have investigated hypotonic tear substitutes^4,7,9,10^ and reported enhanced effects on the tear film stability and ocular surface integrity.

However, those studies indirectly evaluated the effect of hypotonic tear substitutes using other clinical assessments instead of direct changes in tear osmolarity. Despite the technical challenges of measuring osmolarity, the TearLab osmolarity system (OcuSense, Inc., San Diego, CA, US), a point-of-care device^1,11^, allows assessment of tear osmolarity in a quick and simple way.

The purpose of this study was to investigate short-term changes in tear osmolarity of dry eye patients after using artificial tears containing sodium hyaluronate (SH) at different osmolarities.

## Results

Eighty patients with dry eye were included in this study. The mean age of the participants was 51.4 ± 14.2 years, and 70 (87.5%) were female. Table 1 summarizes the demographics and characteristics of the four groups at baseline. There were no statistically significant differences in age, TBUT, corneal staining score, or tear osmolarity between groups before the treatment.

**Table 1:** Patient demographics and characteristics at baseline.

	Group 1 (0.1% isotonic)	Group 2 (0.15% isotonic)	Group 3 (0.3% isotonic)	Group 4 (0.18% hypotonic)	p-value
Age, years	52.1 ± 16.7	52.9 ± 12.3	51.6 ± 11.9	49.0 ± 16.2	0.828
TBUT, sec	3.03 ± 0.96	2.72 ± 0.69	3.00 ± 0.64	3.35 ± 2.33	0.448
NEI score	3.40 ± 3.92	3.60 ± 2.76	2.60 ± 2.47	3.35 ± 2.33	0.560
TOSM, Osm/L	311.60 ± 14.98	309.75 ± 10.78	311.70 ± 16.86	310.50 ± 11.97	0.700

*TBUT* tear break up time, *NEI* National Eye Institute grading system, *TOSM* tear osmolarity, Groups 1–4: isotonic 0.1%, isotonic 0.15%, isotonic 0.3%, and hypotonic 0.18% sodium hyaluronate eyedrops, respectively.

*Statistically significant differences (p < 0.05) according to a Kruskal–Wallis test.

### Changes in tear osmolarity over time after the treatment

**Figure 1:**
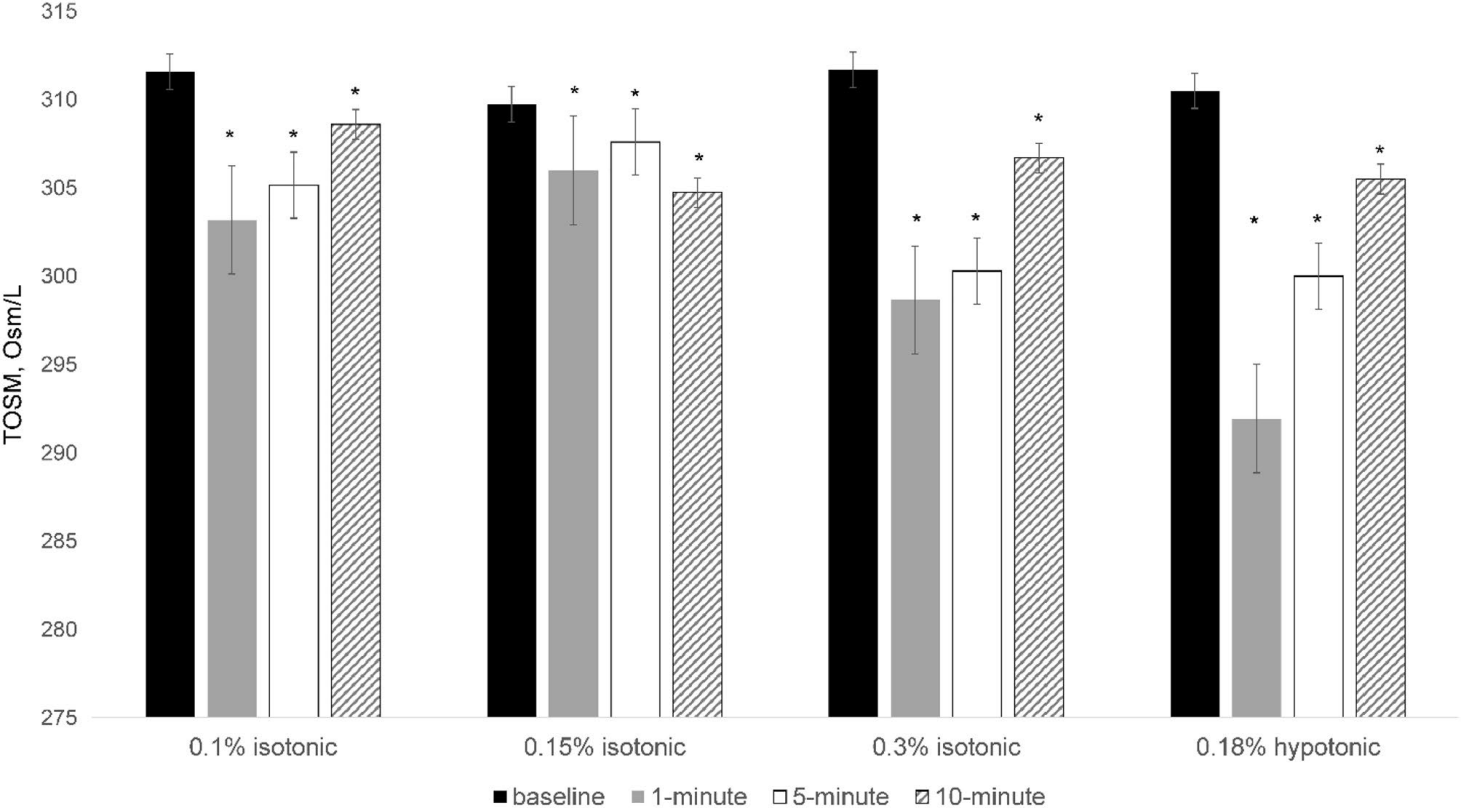
Changes in tear osmolarity over time in each group after the treatment. Bar plot illustrating the mean and standard deviation (error bars). An asterisk (*) indicates a significant change compared with tear osmolarity before treatment in the same group. *TOSM* tear osmolarity. *Statistically significant differences (p < 0.05) according to a repeated-measures ANOVA.

**Figure 2:**
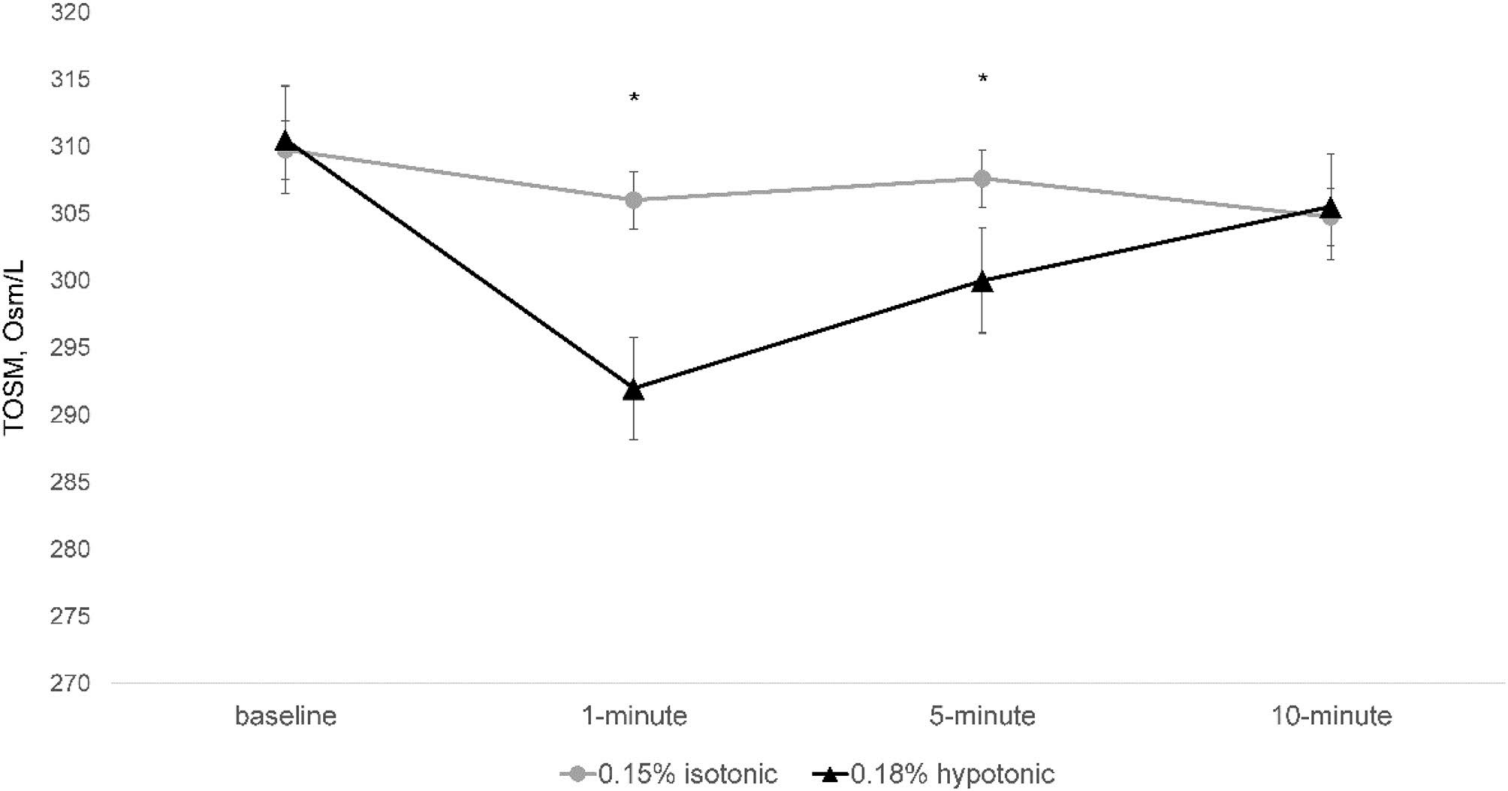
Comparison of isotonic 0.15% and hypotonic 0.18% sodium hyaluronate eye drops in the changes of tear osmolarity after instillation. Line graph illustrating the mean and standard deviation (error bars). *TOSM* tear osmolarity. *Statistically significant differences (p < 0.05) according to a T-test.

**Figure 3:**
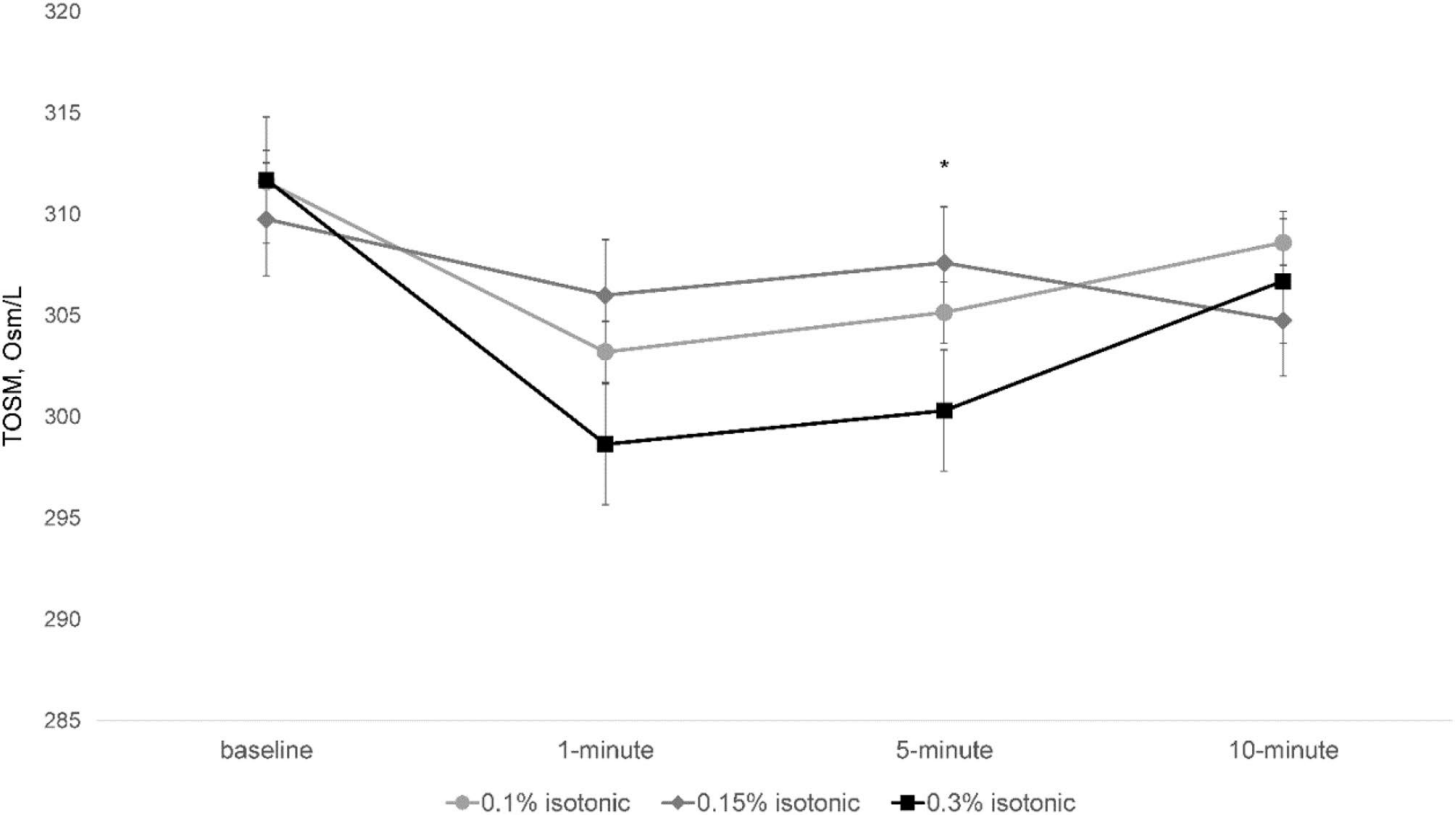
Comparison of three isotonic eye drops containing different sodium hyaluronate concentrations in the changes of tear osmolarity after instillation. Line graph illustrating the mean and standard deviation (error bars). *TOSM* tear osmolarity. *Statistically significant differences (p < 0.05) according to a one-way ANOVA.

All four SH eye drops showed a significant decrease in tear osmolarity at all time points (Fig. 1). This decrease was maximized at 1 min after instillation except for Group 2, the 0.15% isotonic group. When comparing patients from the 0.18% hypotonic group to those in the 0.15% isotonic group, the former showed an enhanced decrease in tear osmolarity at 1 min (T-test, p < 0.001) and 5 min (T-test, p = 0.006) post instillation, but this difference diminished at 10 min (T-test, p = 0.836; Fig. 2). In addition, among the isotonic SH groups at different concentrations, Group 3, the 0.3% isotonic group, showed an enhanced decrease in tear osmolarity after 5 min (one-way ANOVA test, p = 0.009; Fig. 3).

### Average tear osmolarity at 5 and 10 min

At the 5-min time point, the average tear osmolarity of all patients was 302 mOsm/L. This value was used to divide the patients into two groups of higher than vs. lower than 302 mOsm/L regardless of the type of eye drops used. Table 2 summarizes the subgroup analysis. The baseline TBUT was significantly longer in the low tear osmolarity group (mean TBUT: 3.08 ± 0.84 s) compared to the high tear osmolarity group (mean TBUT: 2.72 ± 0.71 s; Mann–Whitney test, p = 0.042). The baseline NEI score was also lower in the low tear osmolarity group (mean NEI score: 2.36 ± 3.14) compared to the high tear osmolarity group (mean NEI score: 3.95 ± 2.51; Mann–Whitney test, p = 0.014) (Table 2). Table 3 summarizes a similar subgroup analysis based on the average value of tear osmolarity at 10 min, which was 306 mOsm/L. The low tear osmolarity group showed significantly lower baseline NEI scores compared to the high tear osmolarity group (Mann–Whitney test, p = 0.043).

**Table 2:** Subgroup analysis based on average tear osmolarity at 5 min (302 mOsm/L).

	TOSM, mOsm/L ≥ 302	TOSM, mOsm/L < 302	p-value
	(n = 44)	(n = 36)	
Age (years)	53.8 ± 12.4	48.4 ± 15.9	0.10
TBUT (s)	2.72 ± 0.71	3.08 ± 0.84	0.042*
NEI score	3.95 ± 2.51	2.36 ± 3.14	0.014*
VAS	1.03 ± 0.90	1.12 ± 0.99	0.669

*TBUT* tear break up time, *NEI* National Eye Institute grading system, *VAS* visual analogue scale, *TOSM* tear osmolarity.

*Statistically significant differences (p < 0.05) according to a Mann–Whitney test.

**Table 3:** Subgroup analysis based on average tear osmolarity at 10 min (306 mOsm/L).

	TOSM, mOsm/L ≥ 306	TOSM, mOsm/L < 306	p-value
	(n = 41)	(n = 39)	
Age (years)	53.4 ± 13.1	49.3 ± 15.2	0.196
TBUT (s)	2.76 ± 0.73	3.02 ± 0.83	0.13
NEI score	3.88 ± 3.03	2.56 ± 2.65	0.043*
VAS	1.08 ± 0.99	1.06 ± 0.88	0.920

*TBUT* tear break up time, *NEI* National Eye Institute grading system, *VAS* visual analogue scale, *TOSM* tear osmolarity.

*Statistically significant differences (p < 0.05) according to a Mann–Whitney test.

### Subjective discomfort

When comparing the subjective discomfort experienced during eye drop instillation, the 0.3% isotonic group showed a higher discomfort score than the 0.1% (Kruskal–Wallis test, p = 0.038) and the 0.15% isotonic (Kruskal–Wallis test, p = 0.028) groups, while the other groups reported no differences.

## Discussion

In 2017, the Tear Film and Ocular Surface Society (TFOS) Dry Eye Workshop (DEWS) II reported that a tear osmolarity value > 308 mOsm/L in either eye or a ≥ 8 mOsm/L difference between eyes was a good indicator of tear film homeostasis and a diseased ocular surface^12^. Several hypotonic eye drops have been commercialized to decrease elevated tear osmolarity, which is believed to be the primary pathogenic factor in dry eye syndrome. In several studies, the effects of hypotonic eye drops have been investigated^4,7,13^, and superior effects on symptoms and corneo-conjunctival conditions have been reported over isotonic eye drops. However, since there are no data on the changes that take place in tear osmolarity, we investigated how isotonic and hypotonic eye drops affect tear osmolarity and the duration of the effect.

In the previous study, the osmolarity values of three isotonic and one hypotonic SH eye drop concentrations were evaluated. The osmolarity was 287 ± 2.08 mOsm/L for 0.1% isotonic SH eye drops, 301.00 ± 2.64 mOsm/L for 0.15% isotonic SH eye drops, and 284.67 ± 1.53 mOsm/L for 0.3% isotonic eye drops. In addition, the osmolarity of the 0.18% hypotonic eye drops was 141.00 ± 1.00 mOsm/L, about half those of the isotonic eye drops^14^.

In the current study, the four SH eye drops all produced a significant decrease in tear osmolarity at all time points up to 10 min after instillation. Tear osmolarity lowering effect of the hypotonic SH solution was lower at 10 min than at 5 min after instillation. In addition, the 0.3% isotonic SH drops showed an enhanced reduction in tear osmolarity at 5 min compared to the other concentrations of isotonic SH eye drops. The longer retention time tendency of 0.3% for SH eye drops may explain this difference^15^.

At the 5- and 10-min time points, the patients were divided into two groups based on mean tear osmolarity. Patients with a higher-than-average tear osmolarity showed worse clinical findings, a shorter TBUT, and greater corneal erosion. The results were consistent with the findings of other studies, which have reported that tear osmolarity has a good correlation with severity of dry eye^1,16^. In patients with severe dry eye syndrome, tear osmolarity may have a higher value at baseline and may return to baseline level quickly after instillation of appropriate SH eye drops.

But, TBUT of patients with a higher-than-average tear osmolarity at 10-min were not statistically significant. It can be related to tear turnover rate (TTR). There is a significant difference in the TTR between symptomatic and asymptomatic subjects. Mean TTR (symptomatic) was 4.89 ± 2.74%/min and mean TTR (asymptomatic) was 11.85 ± 3.31%/min (p < 0.001) ^17^. Even if the TTR of the symptomatic eye is lower, the effect of the SH eye drop may be reduced in 10 min.

This study had a few limitations. As commercially available SH eye drops were used, the concentrations of the 0.15% isotonic and 0.18% hypotonic solutions were slightly different. In addition, patients were not classified according to subtype of dry eye. And this study included only participants with a tear osmolarity of 300 mOsm/L or greater. According to TFOS DEWS II diagnostic criteria, tear osmolarity above 308 mOsm/L is a good indicator of dry eye disease and for the purpose of diagnosing dry eye patients, that criteria would be appropriate. But, the purpose of this study was to find out the difference in change of tear osmolarity according to the osmolarity of instilled SH eyedrops in patients who had already been diagnosed with dry eye. Also, when excluding subjects with an osmolarity of less than 308 mOsm/L, only 14 patients out of 80 were excluded, and the change in osmolarity after instillation was almost same as the results performed based on 300 mOsm/L.

In conclusion, instillation of all types of SH eye drops lowered the tear osmolarity, and the hypotonic SH drops produced an immediate improvement compared to the isotonic SH drops. This difference was observed for 5 min after the instillation, but it diminished after 10 min. Therefore, unless used frequently, any additional lowering effect of hypotonic SH eye drops on tear osmolarity may be limited.

## Methods

This study followed the tenets of the Declaration of Helsinki and was approved by the Institutional Review Board of Korea University Hospital. All patients provided written informed consent prior to beginning the study.

### Subjects and study design

This was a prospective and randomized study conducted from June 2017 to February 2018 that included participants who met the Korean guidelines for dry eye syndrome^18^ and whose tear osmolarity as assessed using TearLab^11^ was 300 mOsm/L or greater. Patients with external ocular disease, glaucoma or other concomitant ocular pathology, a history of ocular surgery, viral or bacterial infection of the cornea and conjunctiva, who regularly wore contact lenses within the past three months, or who reported any current systemic disease that might affect tear film stability were excluded. Only one eye per patient with higher osmolarity was included in this study.

A total of 80 patients was recruited, and they were randomly divided into four groups of 20 patients: Group 1 (isotonic 0.1% SH; Hyalein Mini ophthalmic solution 0.1%, Taejoon, Seoul, Korea), Group 2 (isotonic 0.15% SH; New Hyaluni ophthalmic solution 0.15%, Taejoon, Seoul, Korea), Group 3 (isotonic 0.3% SH; Hyaluni ophthalmic solution 0.3%, Taejoon, Seoul, Korea), and Group 4 (hypotonic 0.18% SH; Kynex2 ophthalmic solution 0.18%, Alcon, Seoul, Korea). Table 4 shows the principal components of SH eye drops.

**Table 4:** Principal components of SH eye drops investigated.

Manufacturer	Solution	Osmolarity	Preservative (%)	Neutralizing agents	Other reported agents (e.g., surfactants, chelating agents and buffers)
Taejoon	Hyalein Mini ophthalmic solution 0.1%	Isotonic	Preservative-free	Hydrochloric acid, Sodium hydroxide	Disodium edetate, Propylene glycol, Aminocaproic acid, Sterile water
Taejoon	New Hyaluni ophthalmic solution 0.15%	Isotonic	Preservative-free	Hydrochloric acid	Tremetamol, Sodium chloride, Sterile water
Taejoon	Hyaluni ophthalmic solution 0.3%	Isotonic	Preservative-free	Hydrochloric acid, Sodium hydroxide	Disodium edetate, Aminocaproic acid, Sodium chloride, Potassium chloride, Sterile water
Alcon	Kynex2 ophthalmic solution 0.18%	Hypotonic	Preservative-free	Magensium chloride, Sodium chloride, Potassium chloride	Disodium edetate, Sodium phosphate monobasic, Borax, Aminocaproic acid, Sterile water

*SH* Sodium hyaluronate.

Clinical evaluation included slit-lamp examination, tear film break up time (TBUT), corneal staining score, and tear osmolarity. All measurements were taken by one ophthalmologist (IHW) in the afternoon (between 12 and 5 p.m.) and the room temperature and humidity were under controlled conditions. Osmolarity was measured first, followed by TBUT and NEI.

The tear osmolarity was assessed using the TearLab system immediately after blinking; the osmometer required a tiny tear sample obtained directly from the inferior tear meniscus using lab-on-chip technology^19^. All tests were performed in the same space using the same devices. Tear osmolarity was evaluated four times for each participant: at baseline and at 1, 5, and 10 min after instillation of the eye drop. The time after instillation of the eye drop was measured with a stopwatch for accuracy. The TBUT was evaluated as follows; after instillation of fluorescein, the participants were asked to blink several times. The time between the last complete blink and the appearance of the first corneal black spot in seconds was measured three times, and the mean value was recorded^20^. The corneal staining score was evaluated using the National Eye Institute scoring method (NEI score) between 0 and 15 ^21^. The subjective discomfort when the drops were instilled was assessed using the visual analogue scale (VAS) from 0 to 10 where 0 means no pain and 10 indicates severe pain or discomfort.

### Statistical analysis

All statistical analyses were performed using IBM SPSS Statistics for Windows, Version 21.0 (IBM Corp., Armonk, NY, USA). First, the Kolmogorov–Smirnov test was used to verify if the examined variables presented a normal distribution. And as a result of the non-normal distribution of the TBUT and NEI score between the four groups of different kinds of SH eyedrops, the Kruskal–Wallis test was used. And to analyze the tear osmolarity at different time points in each group, the repeated measures ANOVA was used. The post-hoc test was performed together (Tukey test) but in this study, two independent groups were compared again through a T-test. A p-value less than 5% was considered to be a statistically significant.

## Data Availability

The raw data for this study are available upon reasonable request from the corresponding author.
